# Rifabutin and Furazolidone Could Be the Candidates of the Rescue Regimen for Antibiotic-Resistant *H. pylori* in Korea

**DOI:** 10.1155/2019/9351801

**Published:** 2019-07-10

**Authors:** Youn I Choi, Sang-Ho Jeong, Jun-Won Chung, Dong Kyun Park, Kyoung Oh Kim, Kwang An Kwon, Yoon Jae Kim, Seol So, Jeong Hoon Lee, Jin-Young Jeong, Sun-Mi Lee

**Affiliations:** ^1^Department of Gastroenterology, Gil Medical Center, Gachon University, Incheon, Republic of Korea; ^2^Department of Gastroenterology, Asan Medical Center, Ulsan University, Seoul, Republic of Korea; ^3^Asan Institute for Life Sciences, Asan Medical Center, University of Ulsan College of Medicine, Seoul 138-736, Republic of Korea

## Abstract

*Background*/*Aim*. In Korea, the rate of *Helicobacter pylori* (*H. pylori*) eradication has declined steadily as a result of increasing resistance to antibiotics, especially dual resistance to clarithromycin and metronidazole. However, microbiological culture data on drug-resistant *H. pylori* is lacking. This study evaluated the antimicrobial efficacy of candidate antibiotics against resistant *H. pylori* strains. *Methods*. After retrospectively reviewing the data from the *Helicobacter* Registry in Gil Medical Center (GMC) and Asan Medical Center (AMC), along with 4 reference strains, we selected the 31 single- or multidrug-resistant strains. The susceptibility of the *H. pylori* strains to seven antibiotics (clarithromycin, metronidazole, levofloxacin, amoxicillin, tetracycline, rifabutin, and furazolidone) and minimum inhibitory concentration were tested using the broth microdilution technique. *Results*. Among 31 antibiotic resistance strains for *H. pylori*, there were no strains resistant to rifabutin or furazolidone, which had MICs of <0.008 and 0.5 *μ*g/mL, respectively. Only one tetracycline-resistant strain was found (MIC < 2 *μ*g/mL). Amoxicillin and levofloxacin were relatively less effective against the *H. pylori* strains compared to rifabutin or furazolidone (resistance rates 22.6%, 1.9%, respectively). Tetracycline showed the relatively low resistance rates (3.2%) for *H. pylori* strains. *Conclusions*. Therefore, along with tetracycline which has already been used as a component for second-line eradication regimen for *Helicobacter*, rifabutin and furazolidone, alone or in combination, could be used to eradicate antibiotic-resistant *H. pylori* strains where drug-resistant *Helicobacter* spp. are increasing.

## 1. Introduction

*Helicobacter pylori* infection is responsible for the development of chronic atrophic gastritis, peptic ulcer disease, and gastric malignant neoplasms such as gastric adenocarcinoma and mucosa-associated lymphoid tissue lymphoma [1, 2]. *H. pylori* is recognized as a Class I carcinogen by the International Agency for Research on Cancer and the World Health Organization [1–3]. The eradication of antibiotic-resistant *H. pylori* is a global health issue [4].

However, multidrug-resistant (MDR) strains of *H. pylori* have been increasing worldwide due to the increased use of antibiotics [1, 2, 4–9]. In Korea, the rate of *H. pylori* eradication has declined steadily in recent decades as a result of increasing resistance to antibiotics, especially dual resistance to clarithromycin and metronidazole [10–12] which has resulted from the increased clinical use of macrolides and metronidazole [13, 14]. In Korea, including Incheon and Seoul where this study was conducted, the rate of resistance of *H. pylori* to clarithromycin has surpassed 15% [15–17]. Several reports suggest that 9.6% of the strains in Korea show dual resistance to clarithromycin and metronidazole [7]. Because the primary failure rate of *H. pylori* eradication has been increasing [12, 13, 18, 19], real-world antimicrobial resistance data are needed to improve therapeutic outcomes. However, there are little recent data on *in vitro* antimicrobial effectiveness in Korea. Indeed, there is no consensus on the optimal rescue therapy for second-line eradication failure. Although the Maastricht V consensus recommended fluoroquinolone-containing therapy as first- or second-line treatment after failure of triple or nonbismuth quadruple therapy, this cannot be applied in Korea because of the increased rate of quinolone resistance [20]. Therefore, we conducted this real-world updated analysis of the *in vitro* antibacterial efficacy against MDR *H. pylori*.

The 2013 revision of the Korean Clinical Practice Guideline for *H. pylori* recommends triple therapy with a proton pump inhibitor (PPI), amoxicillin, and clarithromycin or a bismuth-based quadruple regimen if clarithromycin resistance is suspected [10, 21]. With the failure of first-line therapy, bismuth-based quadruple therapy or a regimen including two or more other antibiotics could be considered [21]. Although levofloxacin- and rifabutin-based triple therapy have been suggested for rescue therapy, there is no consensus on their use in Korea. Therefore, it is necessary to identify antibiotics effective against antibiotic-resistant *H. pylori* [22]. The Maastricht V/Florence guideline recommends culturing *H. pylori*, testing for antimicrobial susceptibility and selecting antibiotics based on the results of resistance tests [23]. However, there are limited data on antimicrobial agents that are effective against antibiotic-resistant *H. pylori* in Korea.

Therefore, this study investigated the antimicrobial activity of rifabutin, furazolidone, and other antibacterial agents as candidates for treating antibiotic-resistant *H. pylori* strains especially focusing on the multidrug-resistant *H. pylori*.

## 2. Patients and Methods

### 2.1. Institutional Review Board Approval

The Institutional Review Boards of Gil Medical Center (GMC) and Asan Medical Center (AMC) reviewed the study protocol (certification number: GAIRB2016-329).

### 2.2. Patient Characteristics

This study examined 4 reference strains and 31 strains isolated from patients at GMC (*n*=15) and AMC (*n*=16) in 2016. We retrospectively reviewed the data of cultures for *Helicobacter pylori* (*H. pylori*) up to 2016 from *Helicobacter pylori* Registry in GMC and AMC. We analyzed and tested the candidate helicobacter antibiotics including amoxicillin, clarithromycin, metronidazole, levofloxacin, and tetracycline from the strains of *H. pylori*. Patients' clinical data such as initial presentation of symptoms, reasons for endoscopy, antibiotics uses history, and reasons for cultures of *H. pylori* were retrospectively reviewed in GMC and AMC. Culture reasons for *Helicobacter* spp. for patients were as follows: (1) patients who have reported several antibiotics experiences or admission to hospital histories in 3 years, (2) first-line or second-line treatment failures, and (3) other clinically suspected medical condition of drug resistance, such as patients with old age more than 65 years who have had more chance to exposure into several antibiotics, or patients with severe comorbid conditions such as congestive heart failure, liver cirrhosis, renal failure, autoimmune disorders, pulmonary disease, and so on.

### 2.3. *H. pylori* Strain Isolation

Mucosal tissues collected from the gastric antrum of each patient were used to isolate *H. pylori*. To isolate the bacteria, the tissues were placed in an aseptic Petri dish, then crushed using a surgical knife and cultivated in Brucella broth agar, and supplemented with 5% sheep blood containing vancomycin (10 *μ*g/mL), trimethoprim (5 *μ*g/mL), amphotericin B (5 *μ*g/mL), and polymyxin B (2.5 IU). These were cultured at 37°C under microventilation conditions (5% O_2_, 10% CO_2_, and 85% N_2_). The colonies obtained from the initial cultures were confirmed to be *H. pylori* using Gram staining and biochemical methods. Each strain identified as *H. pylori* was stored at −70°C in Brucella liquid medium (Difco Laboratories, Detroit, MI, USA) containing 15% glycerol. Shortly before the subsequent experiments, they were melted, multiplied, and used.

### 2.4. *H. pylori* Antimicrobial Susceptibility Testing

The minimum inhibitory concentrations (MICs) of the following antimicrobial agents were tested: clarithromycin (Abbott Laboratories, Abbott Park, IL, USA), amoxicillin, metronidazole, tetracycline, levofloxacin, rifabutin, and furazolidone (all from Sigma Chemical Co., St. Louis, MO, USA).

### 2.5. Culture Conditions

To test the MICs of *H. pylori*, we used the agar dilution method recommended by the Clinical and Laboratory Standards Institute (CLSI) [24], an internationally recognized antimicrobial susceptibility testing laboratory, using Mueller–Hinton agar (Difco Laboratories, Detroit, MI, USA) supplemented with 5% defibrinated sheep blood. The medium was sterilized by autoclaving, and each antimicrobial agent was serially diluted in medium supplemented with 5% sheep blood (Comed, Seoul, Korea), which was collected within 2 weeks of birth and cooled to 80°C. Then, the suspension of *H. pylori* strains (1 × 10^7^ colony-forming units) was cultured in blood culture medium for 72 hours and inoculated on Mueller–Hinton agar containing an antimicrobial agent using a micropipette. This was incubated at 37°C for 3 days under microventilation conditions, and the presence of bacterial colonies was observed. Each experiment was performed in triplicate, and experiments were repeated at least three times per strain.

### 2.6. Antimicrobial Resistance Criteria

The MIC was defined as the minimum dilution concentration of the antimicrobial agent that did not produce bacterial colonies. The criterion for resistance to each antimicrobial agent was set to MIC >1 *μ*g/mL, as given in the CLSI for resistance to clarithromycin [24, 25]. The resistance criteria for antimicrobial agents were set to greater than 0.5 *μ*g/mL for amoxicillin [7], 8 mg/mL for metronidazole [7], 4 *μ*g/mL for tetracycline [7], 1 *μ*g/mL for levofloxacin antibiotics [26], 0.25 *μ*g/mL for rifabutin [7, 27], and 4 *μ*g/mL for furazolidone [7, 28]. Resistance to two or more antimicrobials was defined as multidrug resistance (MDR) [29]. For quality control, *H. pylori* strain (ATCC 43504), which is used as a standard strain in CLSI, was selected [24].

## 3. Results

### 3.1. Characteristics of the Study Population

The mean age of the study population was 58.2 ± 10.3 years, and 41.9% (*n*=13) were more than 65 years. Reasons for endoscopy of study populations were as follows: (1) patients who received endoscopy for routine health checkup (*n*=15) or (2) patients with gastrointestinal symptoms such as dyspepsia, regurgitation, or pain (*n*=16). The most common reason for eradication was peptic ulcer disease (*n*=15, 48.4%) (Table 1).

### 3.2. Characteristics of the Isolated Strains of *H. pylori*

Of the 31 isolated strains, 9 were resistant to one antimicrobial agent and 22 (71.0%) were resistant to two or more antimicrobial agents, including 13 strains resistant to two antibiotics (41.9%), seven strains resistant to three antibiotics (22.6%), and two strains resistant to four antibiotics (6.4%). The most common combination of drug resistance was clarithromycin + metronidazole (16 strains, 51.6%) (Table 2).

Of the 31 strains, 1 (3.2%) was resistant to tetracycline and none were resistant to rifabutin or furazolidone (Table 3).

### 3.3. Clarithromycin MIC and Resistance in *H. pylori* Strains

**Figure 1:**
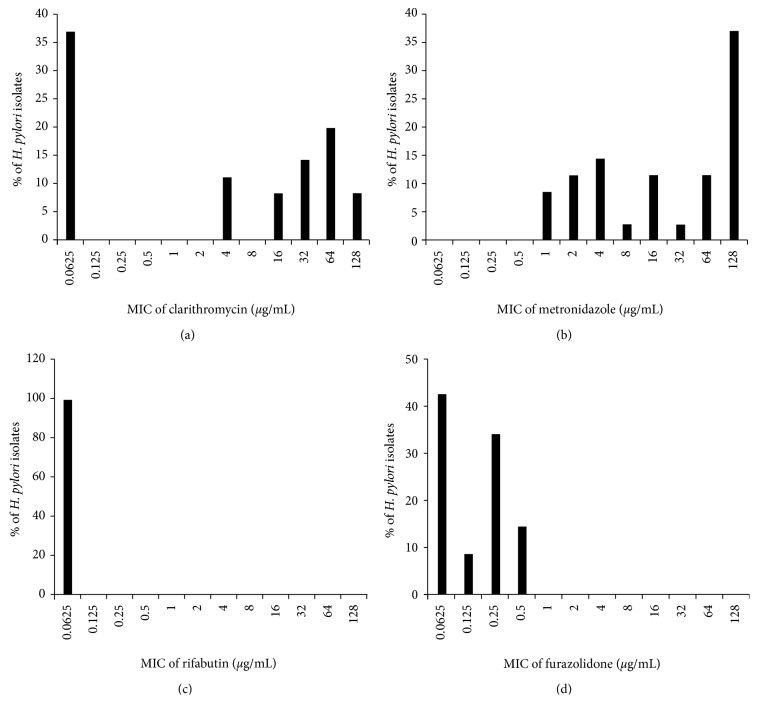
MIC distribution of (a) clarithromycin, (b) metronidazole, (c) rifabutin, and (d) furazolidone for *H*. *pylori*. MIC, minimum inhibitory concentration.

The range of MICs for clarithromycin was very broad, from 0.03 to >128 *μ*g/mL. Overall, the MICs of the 31 strains had two distinct peaks (Figure 1), with MIC < 0.0625 *μ*g/mL in 37.1% of the isolates and very high MICs in others (e.g., 16, 32, 64, and 128 *μ*g/mL). The CLSI criterion for clarithromycin resistance is MIC >1 *μ*g/mL. [24] Of the 31 strains tested, 22 were resistant (71.1%) according to this criterion (Table 3).

### 3.4. Metronidazole MIC and Resistance in *H. pylori* Strains

The MIC for metronidazole ranged from 1 to 128 *μ*g/mL (Figure 1). The resistance standard for metronidazole was established to exceed 8 *μ*g/mL, which is normally used without established criteria [24]. The resistance rate according to this standard was 67.7% (21/31).

### 3.5. Simultaneous Clarithromycin and Metronidazole Resistance in *H. pylori* Strains

Of the 31 strains studied, 22 showed MDR and 16 strains (51.6%) were resistant to both clarithromycin and metronidazole, accounting for 64% of all MDR strains. Rifabutin and furazolidone had excellent antibacterial activity with no resistant strains.

### 3.6. Quinolone MIC and Resistance in *H. pylori* Strains

The MIC for levofloxacin ranged from 0.25 to 64 *μ*g/mL. The criterion for bacterial resistance to quinolone antibiotics is MIC >1 *μ*g/mL [24]. Using this standard, 41.9% (13/31) of the strains were resistant to levofloxacin (Table 3).

### 3.7. Tetracycline MIC and Resistance in *H. pylori* Strains

The MIC for tetracycline ranged from <0.03 to 2 *μ*g/mL (Figure 1). Only 1 of the 31 strains was resistant to tetracycline.

### 3.8. Rifabutin and Furazolidone MIC and Resistance in *H. pylori* Strains

The MIC for rifabutin ranged from <0.00098 to 0.0078 *μ*g/mL and that of furazolidone from <0.03 to 0.5 *μ*g/mL (Figure 1). The criteria for resistance are MIC >0.25 *μ*g/mL for rifabutin and MIC >4 *μ*g/mL for furazolidone. None of the 31 strains was resistant to either drug.

## 4. Discussion

In this *in vitro* analysis of antimicrobial effectiveness, we aimed to investigate which of the antibiotics were effective for multidrug resistance *H. pylori* and found that rifabutin and furazolidone had excellent potential for eradicating not only single-drug-resistant *H. pylori* but also MDR *H. pylori* through culture-based data in Korea. No strains were resistant to rifabutin or furazolidone, which had very low MICs of <0.00098 and 0.5 *μ*g/mL, respectively, for all strains. Tetracycline also had low MICs, which were <2 *μ*g/mL for all but one resistant strain. Therefore, along with tetracycline which has already been used as a component for second-line eradication regimen for *Helicobacter*, rifabutin and furazolidone, alone or in combination, could be used to eradicate antibiotic-resistant *H. pylori* strains. In comparison, amoxicillin and levofloxacin were only partially effective against the *H. pylori* strains in this *in vitro* study.

To our knowledge, this is the first *in vitro* antimicrobial analysis of antibiotics candidate, rifabutin, and furazolidone, in MDR *H. pylori* in Korea where clarithromycin resistance rate exceeds 15%.

Studies have explored several antibiotics as rescue therapy following the failure of first- and second-line treatment in Korea. Sung et al. evaluated the efficacy of rifabutin-based rescue therapy among patients with third-, fourth-, or fifth-line eradication failure [30]. Rifabutin-based rescue therapy had an approximately 55% eradication rate with few side effects. Jeong et al. reported that rifabutin-based therapy eradicated over 70% of *H. pylori* in third-line rescue therapy in 21 patients [31]. There might be several reasons for the variation in the eradication rate of rifabutin-based therapy in Korea. First, the sample sizes of these studies were small. Second, because *H. pylori* eradication failure is diagnosed using the rapid urease test after treatment, the causes of eradication failure were unclear. Major causes of eradication failure apart from drug-resistant *H. pylori* are loss of compliance with treatment, the density of *H. pylori* in the stomach wall, presence of CagA, and smoking. In our *in vitro* antibacterial efficacy analysis of drug-resistant *H. pylori*, rifabutin showed excellent antimicrobial activity in MDR *H. pylori*. To our knowledge, this is the first *in vitro* analysis of rifabutin in MDR *H. pylori*. Given the high rates of tuberculosis infection and antituberculosis medication use in Korea, the low rate of rifabutin-resistant *Helicobacter* spp. is interesting. Before selecting rifabutin as rescue therapy in Korea, clinicians should carefully monitor its major side effects, including rare myelosuppressive events [27, 30, 32–34], strictly limit its use to confirmed eradication cases, and monitor patient compliance closely to avoid the development of rifamycin-resistant tuberculosis [32].

Another option for third-line rescue therapy in Korea is furazolidone. In a meta-analysis, Mohammadi et al. reported an *H. pylori* eradication rate exceeding 80% and a low rate of side effects in Iran, where MDR *H. pylori* is common [35]. In a multicenter randomized controlled trial in China, where MDR *H. pylori* is also common, Xie et al. reported an eradication rate with furazolidone of up to 90% in 720 patients with *H. pylori* [36]. Despite limited data on furazolidone as a *Helicobacter* spp. treatment in Korea, Kim et al. reported a 1.5% resistance rate in first-line treatment failure patients [37]. However, their study was conducted in 2001 and recent data on furazolidone for *Helicobacter* spp. eradication in Korea are not available. Because the antibacterial resistance rate differs among countries, large multicenter population-based studies are needed in Korea. Our study showed that furazolidone has an extremely low rate of resistance in drug-resistant *H. pylori in vitro*.

Tetracycline is one component of bismuth-based quadruple therapy (PPI, bismuth, metronidazole, and tetracycline), which is effective for *Helicobacter* spp. eradication, especially in areas with high levels of clarithromycin resistance [13, 38]. In our *in vitro* study, only one strain of drug-resistant *H. pylori* was resistant to tetracycline, suggesting that tetracycline is still effective for drug-resistant *H. pylori* eradication in Korea.

This study had several limitations. First, it did not confirm the eradication rate by actually treating the patients, so it is impossible to know how the *in vitro* results will correspond to *in vivo* effects. Further studies need to confirm the eradication rate of furazolidone and rifabutin and safety in actual patients. Second, because we studied antibiotic-resistant *H. pylori* strains that were selected randomly, our result might not reflect the general prevalence of antibiotic-resistant *H. pylori* in Korea; selection bias could be an issue. Nevertheless, this was the first study of the effectiveness of rifabutin and furazolidone in Korea though *H. pylori* culture data, and almost all of the antibiotics used in clinical practice for *H. pylori* eradication in Korean were covered. Third, because the antibiotic resistance data for *H. pylori* were relatively small in this study, it should be cautious for physicians to generalize these results to a general population or other ethics. Given that the cost and time to obtain results of MIC for each antibiotic from culture data of *Helicobacter* spp., it might be important to invent and use molecular methods to evaluate the resistance of drugs directly in biopsies samples when it is impossible to isolate the strains [39].

In conclusion, this study showed that rifabutin, furazolidone, and tetracycline, alone or in combination, are promising candidates for rescue therapy of antibiotic-resistant *H. pylori* strains, as no definitive rescue therapy for *H. pylori* eradication is available. A future eradication regimen could potentially be designed based on these results.

## Figures and Tables

**Table 1:** The demographic characteristics of the patient with *H. pylori* strain (*N*=31).

Characteristics	*N* (%)
Age, mean ± SD (years)	58.2 ± 10.3
Age >65 years, *N* (%)	13 (41.9%)
Men, *N* (%)	16 (51.6%)
Smoking, *N* (%)	6 (19.3%)
Drinking, *N* (%)	12 (38.7%)
Comorbidity	
Diabetes mellitus type 2	2 (6.4%)
Hypertension	8 (25.8%)
Liver cirrhosis	1 (3.2%)
Cerebrovascular disorders	1 (3.2%)
Thyroid cancer	1 (3.2%)
Idiopathic pulmonary fibrosis	1 (3.2%)
Pulmonary tuberculosis	1 (3.2%)
Reasons for *Helicobacter* spp. cultures^§^	
First-line or second-line empirical treatment failure	9 (29.0%)
Patients' reported antibiotic uses history in 3 years	15 (48.4%)
Other clinically suspected medical condition of drug resistance^**¶**^	13 (41.9%)
Reason for eradication for *H. pylori*	
Peptic ulcer disease	15 (48.4%)
Early gastric cancer	2 (6.5%)
MALToma	4 (12.9%)
Atrophic gastritis	10 (32.3%)

^¶^Other clinically suspected medical conditions of drug resistance; patients with old age more than 65 years who have had more chance to exposure into several antibiotics or patients with severe comorbid conditions such as congestive heart failure, liver cirrhosis, renal failure, autoimmune disorders, pulmonary disease, and so on. ^§^Total sum of population is not 100% since duplication cases. NSAID, nonsteroidal anti-inflammatory drug; HTN, hypertension; PUD, peptic ulcer disease; EGC, early gastric cancer; MALToma, mucosa-associated lymphoid tissue lymphoma.

**Table 2:** Drug resistance of *H. pylori* isolates (*N*=31).

No. of resistant antibiotics	Types of drug resistance	No. of strains (%)
1	CLR	3 (9.7%)
	MT	3 (9.7%)
	LVX	3 (9.7%)

2	AMX + CLM	2 (6.5%)
	AMX + LVX	1 (3.2%)
	CLM + MET	10 (32.3%)

3	CLM + MET + LVX	4 (12.9%)
	AMX + MET + LVX	2 (6.5%)
	AMX + CLM + LVX	1 (3.2%)

4	CLM + MET + TET + LVX	1 (3.2%)
	AMX + CLM + MET + LVX	1 (3.2%)

Total	—	31

AMX, amoxicillin; CLM, clarithromycin; MET, metronidazole; TET, tetracycline; LVX, levofloxacin.

**Table 3:** Prevalence of antibiotic resistance of *H. pylori* isolates.

	Resistant breakpoint of MIC (*μ*g/mL)	No. of resistant strains/Total strains	Resistance rate (%)
Clarithromycin	>1	22/31	71.1
Metronidazole	>8	21/31	67.7
Levofloxacin	>1	13/31	41.9
Amoxicillin	>0.5	7/31	22.6
Tetracycline	>4	1/31	3.2
Rifabutin	>0.25	0/31	0
Furazolidone	>4	0/31	0

MIC, minimum inhibitory concentration.

## Data Availability

The data used to support the findings of this study are available from the corresponding author upon request.

## References

[1] Savoldi A., Carrara E., Graham D. Y., Conti M., Tacconelli E. (2018). Prevalence of antibiotic resistance in *Helicobacter pylori*: a systematic review and meta-analysis in world health organization regions. *Gastroenterology*.

[2] Kekilli M., Onal I., Ocal S., Dogan Z., Tanoglu A. (2016). Inefficacy of triple therapy and comparison of two different bismuth-containing quadruple regimens as a firstline treatment option for *Helicobacter pylori*. *Saudi Journal of Gastroenterology*.

[3] Fasciana T. C. G., Capra C., Zambuto S. (2017). *Helicobacter pylori* and epstein-barr co-infection in gastric disease. *Pharmacologyonline*.

[4] Pellicano R., Zagari R. M., Zhang S., Saracco G. M., Moss S. F. (2018). Pharmacological considerations and step-by-step proposal for the treatment of *Helicobacter pylori* infection in the year 2018. *Minerva Gastroenterologica e Dietologica*.

[5] Kuo Y.-T., Liou J.-M., El-Omar E. M. (2017). Primary antibiotic resistance in *Helicobacter pylori* in the asia-pacific region: a systematic review and meta-analysis. *Lancet Gastroenterology & Hepatology*.

[6] Hu Y., Zhu Y., Lu N.-H. (2017). Primary antibiotic resistance of *Helicobacter pylori* in China. *Digestive Diseases and Sciences*.

[7] Chung J. W., Kim S. Y., Park H. J., Chung C. S., Lee H. W., Lee S. M. (2017). In vitro activity of diphenyleneiodonium toward multidrug-resistant *Helicobacter pylori* strains. *Gut and Liver*.

[8] Malfertheiner P., Megraud F., O’Morain C. A. (2017). Management of *Helicobacter pylori* infection-the maastricht v/florence consensus report. *Gut*.

[9] Fasciana T., Cala C., Bonura C., Di Carlo E., Matranga D., Scarpulla G. (2015). Resistance to clarithromycin and genotypes in *Helicobacter pylori* strains isolated in Sicily. *Journal of Medical Microbiology*.

[10] Lee S. W., Kim H. J., Kim J. G. (2015). Treatment of *Helicobacter pylori* infection in Korea: a systematic review and meta-analysis. *Journal of Korean Medical Science*.

[11] Auesomwang C., Maneerattanaporn M., Chey W. D., Kiratisin P., Leelakusolwong S., Tanwandee T. (2018). Ten-day high-dose proton pump inhibitor triple therapy versus sequential therapy for *Helicobacter pylori* eradication. *Journal of Gastroenterology and Hepatology*.

[12] Kim J. S., Kim B. W., Hong S. J., Kim J. I., Shim K. N., Kim J. H. (2016). Sequential therapy versus triple therapy for the first line treatment of *Helicobacter pylori* in Korea: a nationwide randomized trial. *Gut and Liver*.

[13] Gisbert J. P. (2009). Review: second-line rescue therapy of helicobacter pylori infection. *Therapeutic Advances in Gastroenterology*.

[14] Ko S. W., Kim Y. J., Chung W. C., Lee S. J. (2019). Bismuth supplements as the first-line regimen for *Helicobacter pylori* eradication therapy: systemic review and meta-analysis. *Helicobacter*.

[15] Lee J. W., Kim N., Kim J. M., Nam R. H., Chang H, Kim J. Y. (2013). Prevalence of primary and secondary antimicrobial resistance of *Helicobacter pylori* in Korea from 2003 through 2012. *Helicobacter*.

[16] Chung J. W., Jung Y. K., Kim Y. J. (2012). Ten-day sequential versus triple therapy for *Helicobacter pylori* eradication: a prospective, open-label, randomized trial. *Journal of Gastroenterology and Hepatology*.

[17] Chung J. W., Lee G. H., Jeong J. Y., Lee S. M., Jung J. H., Choi K. D. (2012). Resistance of *Helicobacter pylori* strains to antibiotics in Korea with a focus on fluoroquinolone resistance. *Journal of Gastroenterology and Hepatology*.

[18] Fujioka T., Aoyama N., Sakai K., Miwa Y., Kudo M., Kawashima J. (2012). A large-scale nationwide multicenter prospective observational study of triple therapy using rabeprazole, amoxicillin, and clarithromycin for *Helicobacter pylori* eradication in Japan. *Journal of Gastroenterology*.

[19] Chey W. D., Leontiadis G. I., Howden C. W., Moss S. F. (2017). ACG clinical guideline: treatment of *Helicobacter pylori* infection. *American Journal of Gastroenterology*.

[20] Lee H., Hong S. N., Min B. H., Lee J. H., Rhee P. L., Lee Y. C. (2015). Comparison of efficacy and safety of levofloxacin-containing versus standard sequential therapy in eradication of *Helicobacter pylori* infection in Korea. *Digestive and Liver Disease*.

[21] Kim S. G., Jung H. K., Lee H. L., Jang J. Y., Lee H., Kim C. G. (2014). Guidelines for the diagnosis and treatment of *Helicobacter pylori* infection in Korea, 2013 revised edition. *Journal of Gastroenterology and Hepatology*.

[22] Jonaitis L., Pellicano R., Kupcinskas L. (2018). *Helicobacter pylori* and nonmalignant upper gastrointestinal diseases. *Helicobacter*.

[23] Suzuki H., Matsuzaki J. (2018). *Helicobacter pylori* eradication failure may have confounded the recent large-scale health database study that showed proton pump inhibitors increase gastric cancer risk. *Gut*.

[24] Midolo P. D., Bell J. M., Lambert J. R., Turnidge J. D., Grayson M. L. (1997). Antimicrobial resistance testing of *Helicobacter pylori*: a comparison of etest and disk diffusion methods. *Pathology*.

[25] Heep M., Beck D., Bayerdorffer E., Lehn N. (1999). Rifampin and rifabutin resistance mechanism in *Helicobacter pylori*. *Antimicrobial Agents and Chemotherapy*.

[26] Akada J. K., Shirai M., Fujii K., Okita K., Nakazawa T. (1999). In vitro anti-*Helicobacter pylori* activities of new rifamycin derivatives, KRM-1648 and KRM-1657. *Antimicrobial Agents and Chemotherapy*.

[27] Fiorini G., Zullo A., Vakil N., Saracino I. M., Ricci C., Castelli V. (2018). Rifabutin triple therapy is effective in patients with multidrug-resistant strains of *Helicobacter pylori*. *Journal of Clinical Gastroenterology*.

[28] Zhuge L., Wang Y., Wu S., Zhao R. L., Li Z., Xie Y. (2018). Furazolidone treatment for *Helicobacter Pylori* infection: a systematic review and meta-analysis. *Helicobacter*.

[29] Bravo D., Hoare A., Soto C., Valenzuela M. A., Quest A. F. (2018). *Helicobacter pylori* in human health and disease: mechanisms for local gastric and systemic effects. *World Journal of Gastroenterology*.

[30] Sung J., Kim N., Park Y. H. (2017). Rifabutin-based fourth and fifth-line rescue therapy in patients with for *Helicobacter pylori* eradication failure. *Korean Journal of Gastroenterology*.

[31] Jeong M. H., Chung J.-W., Lee S. J. (2012). Comparison of rifabutin- and levofloxacin-based third-line rescue therapies for *Helicobacter pylori*. *Korean Journal of Gastroenterology*.

[32] Ciccaglione A. F., Tavani R., Grossi L., Cellini L., Manzoli L., Marzio L. (2016). Rifabutin containing triple therapy and rifabutin with bismuth containing quadruple therapy for third-line treatment of *Helicobacter pylori* infection: two pilot studies. *Helicobacter*.

[33] Mori H., Suzuki H., Matsuzaki J. (2016). Rifabutin-based 10-day and 14-day triple therapy as a third-line and fourth-line regimen for *Helicobacter pylori* eradication: a pilot study. *United European Gastroenterology Journal*.

[34] Lim H. C., Lee Y. J., An B., Lee S. W., Lee Y. C., Moon B. S. (2014). Rifabutin-based high-dose proton-pump inhibitor and amoxicillin triple regimen as the rescue treatment for *Helicobacter pylori*. *Helicobacter*.

[35] Mohammadi M., Attaran B., Malekzadeh R., Graham D. Y. (2017). Furazolidone, an underutilized drug for *H. pylori* eradication: lessons from Iran. *Digestive Diseases and Sciences*.

[36] Xie Y., Zhu Y., Zhou H., Lu Z. F., Yang Z., Shu X. (2014). Furazolidone-based triple and quadruple eradication therapy for *Helicobacter pylori* infection. *World Journal of Gastroenterology*.

[37] Kim J. J., Reddy R., Lee M., Kim J. G., El-Zaatari F. A., Osato M. S. (2001). Analysis of metronidazole, clarithromycin and tetracycline resistance of *Helicobacter pylori* isolates from Korea. *Journal of Antimicrobial Chemotherapy*.

[38] Almeida N., Romaozinho J. M., Donato M. M., Luxo C., Cardoso O., Cipriano M. A. (2014). Triple therapy with high-dose proton-pump inhibitor, amoxicillin, and doxycycline is useless for *Helicobacter pylori* eradication: a proof-of-concept study. *Helicobacter*.

[39] Lorusso F., Caleca M. P., Bellavia C., Pistoia D., Gallina S., Speciale R. (2018). The EBV-DNA can be used as a diagnostic and follow-up parameter of the rhinopharyngeal tumors in the non-endemic population of the western sicily. *Indian Journal of Otolaryngology and Head & Neck Surgery*.

